# Hepatic metastasis in Frantz’s tumor: A case report

**DOI:** 10.1016/j.ijscr.2020.04.037

**Published:** 2020-05-11

**Authors:** Gisela Pereira Xavier Albuquerque, Aline Maria Pereira Cruz Ramos, Ana Karyssa Mendes Anaissi, Samia Demachki, Williams Fernandes Barra, Helena Cecilia Branches Soares, Marta Solange Camarinha Ramos Costa, Amanda Carolina Rozario Pantoja, Geraldo Ishak, Paulo Pimentel Assumpção

**Affiliations:** a University Hospital João de Barros Barreto/Ebserh - Brazilian Company of Hospital Services, Mundurucus Street, N.4487, Guamá, Belém - PA, 66073-000, Belém, PA, Brazil; b Nursing School, Federal University of Pará, Augusto Corrêa Street, Belém, PA, CEP: Cep: 66075-110, Brazil; c Research Center on Oncology, Institute of Health Sciences, Federal University of Pará, Mundurucus Street, N.4487, Guamá, Belém - PA, 66073-000, Belém, PA, Brazil

**Keywords:** SPN, solid pseudopapillary neoplasm, WHO, World Health Organization, ACT, abdomen computed tomography, TA-USG, total abdominal ultrasound, MR, magnetic resonance, CT, computed tomography, AJCC, American Joint Committee on Cancer, STP, solid pseudopapillary tumor of pancreas, Solid pseudopapillary neoplasm, Frantz’s tumor, Pancreatic tumor, Hepatic metastasis, Case report

## Abstract

**Introduction:**

Frantz’s Tumor or Solid Pseudopapillary Neoplasm (SPN) is rare with a solid-cystic pattern, and most common in young women.

**Presentation of case:**

This study based on guidelines for case reports (SCARE) reports a case of SPN in a teenager aged 13 years at the diagnosis time, attended at a teaching public hospital in Brazil, which evolved into liver metastases. Clinical, laboratory, therapeutic and imaging data were collected from the physical chart and analyzed in light of current publications on the topic. The first consultation occurred in January 2012, where weight loss, fever, vomiting, left-sided hypochondrium and epigastric pain were reported. Imaging exams evidenced an expansive heterogeneous process in the pancreatic tail; however, laboratory exams and tumor markers did not present alteration in relation to reference values. In March of 2012, she underwent caudal body pancreatectomy, splenectomy, segmental colectomy and colo-coloanastomosis as a function of the intraoperative findings. After 63 months, right-sided hepatectomy was performed to resect metastases. Currently, she is undergoing outpatient monitoring, without complaint or alterations in imaging and laboratory exams, totaling 100 months of global survival.

**Discussion:**

This is an interesting case report of a rare tumor, in so far as without any adjuvant chemotherapies, an 8-year long survival time could be achieved in this particular type of tumor despite initially large tumor expansion and later liver metastases.

**Conclusion:**

Additional epidemiological studies, molecular and clinical trials are required to increase knowledge on SPN.

## Highlights

• This is a borderline tumor with indolent biological behavior.
• There are still multiple poorly understood factors that can dictate its malignant transformation and prognosis.
• Here, a rare tumor whose age and survival are tangent the literature reports describe.
• There is a need for a long and rigorous segment to identify possible long-term metastases.

## 1 Introduction

Frantz’s Tumor was initially described by Virginia Kneeland Frantz in 1959, since 1996, it has been denominated Solid Pseudopapillary Neoplasm (SPN) according to its histogenesis [1]. It is a rare tumor with an incidence of 1–2% among exocrine tumors of the pancreas most common in young women [2,3].

The SPN is solitary tumor, well circumscribed and encapsulated, that presents a solid-cystic pattern, necrosis, hemorrhage and cystic degeneration [4,5]. According to the World Health Organization (WHO), it is a solid pseudopapillary neoplasm, with morphological classification M-8452/1, with boderline features [6].

Although the malignancy criteria are still not clearly, it occurs in about 15% of the cases, with deep invasion of the pancreatic parenchyma, perineural, angioinvasion or distant metastasis (liver, peritoneal and lymph-nodal) [7].

The present study, based on guidelines for case reports (SCARE), aimed to report a case of SPN in a 13-year-old child, attended at a teaching public hospital in the Amazon region of Brazil that evolved with liver metastases, but it has a high overall survival thanks to the surgical treatment performed.

## 2 Presentation of case

**Fig. 1:**
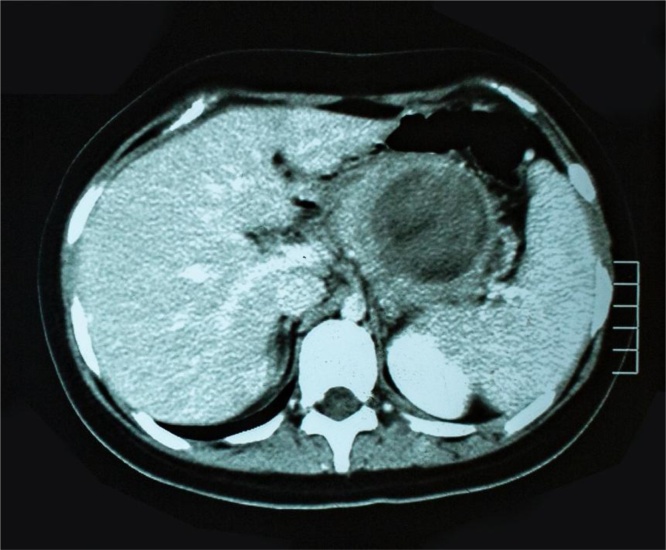
Primary tumour.

A female adolescent, student, aged 13 years, no family history of cancer, realized the first consultation in January of 2012, presented a principal complaint of epigastric and left-hypochondrium pain, weight loss, fever and vomiting. She denies comorbidities, history of alcohol use, tobacco use or cancer in the family. The findings from imaging evidenced a heterogeneous expansive process in the tail of the pancreas (6.5 × 5.6 cm) (Fig. 1). The laboratory exams and tumor markers did not present alterations in relation to the reference values.

**Fig. 2:**
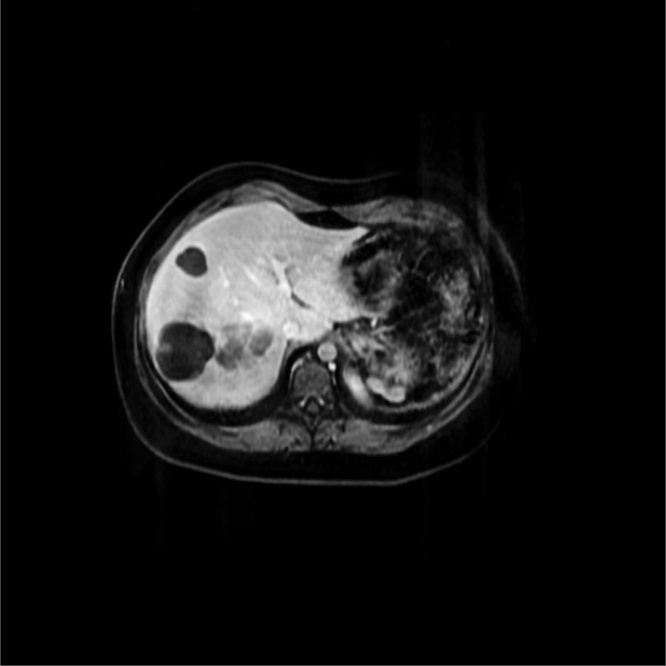
Hepatic metastasis.

In March of 2012, she was submitted to caudal body pancreatectomy, splenectomy, segmental colectomy at 15 cm of the transverse, colo-coloanastomosis and resection of 18 regional lymph nodes (Fig. 2). It should be emphasized that intraoperatively, the surgeon observed macroscopically tumoration of approximately 8 cm occupying the body and tail of the pancreas, adhering to the transverse mesocolon, posterior wall of the stomach, third duodenal portion, and closely related to the spleen; absence of ascites, of peritoneal carcinomatosis and of liver metastasis.

The histopathology and immunohistochemistry concluded the presence of solid pseudopapillary neoplasm, encapsulated, measuring 4.5 × 4.3 × 3.5 cm, with bulging external surface, accentuated cystic alteration, hemorrhage and extensive area of necrosis, with free surgical margins, without evidence of vascular or neural invasion, absence of lymph-nodal metastasis and positive for Vimentin, Cytokeratins (AE1/AE3) and CD10 (CALLA, 56C6).

During 30 postoperative months, the patient reported retrosternal “burning” during outpatient accompaniment, with improvement after the use of pantoprazole. The result of Total Abdominal USG showed the liver with irregular form, wrinkled surface and enlarged dimensions, parenchyma with altered echogenicity and heterogeneous echotexture. The liver vessels did not present alterations and serology was negative for viral hepatites. Then in the 38th month, the patient complained of a frequent, diffuse, slight pain in the abdominal region.

**Fig. 3:**
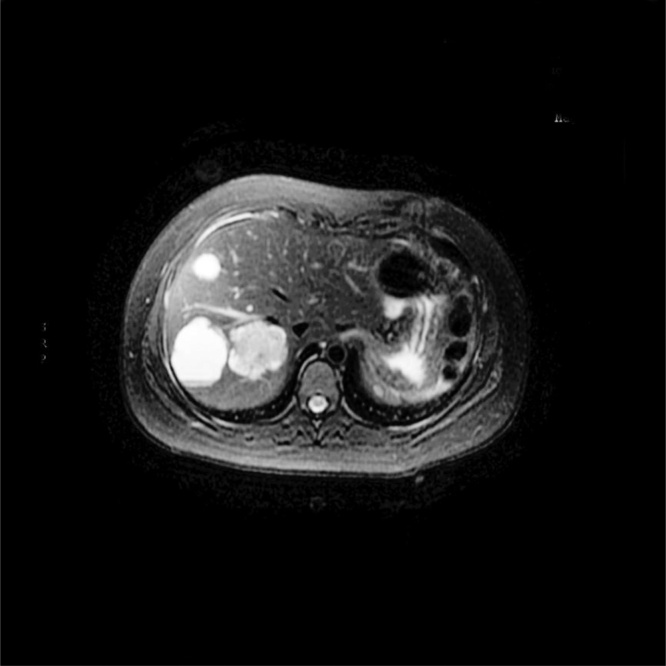
Post resection with liver metastasis.

In the 46th postoperative month, she referenced a sensation of postprandial gastric plenitude and sporadic fever. Abdominal ultrasound showed 4 hypoechoic rounded cystic images with interior trabeculated in segments VI, VII and VIII, measuring between 2 and 2.6 cm. Computed tomography of the abdomen identified the liver of normal size, with heterogeneous attenuations of the parenchyma, the largest of which being 4.8 cm in segment VIII, of lobulated contours and well-defined limits, without significant enhancement after injection of contrast (Fig. 3).

Given the suspicion of liver metastasis, magnetic resonance of the upper abdomen was solicited (performed 50 months after surgery), which evidenced multiple oval encapsulated formations, diffuse in the liver parenchyma, with an aspect that was solid, cystic and non-characteristic, suggesting relation to secondary neoplastic implants. The tumor markers and thoracic CT did not present alterations.

Magnetic resonance was repeated in the 56th postoperative month, and revealed the liver with normal dimensions and contours, but with multiple nodular images, without significant uptake of liver-specific contrast medium, suggestive of cysts (5 cm in segment VIII and 4.6 cm in segment VI, on the major axial axes). Yet another lesion was noted (5 cm in segment VIII), without significant uptake of contrast medium, possibly corresponding to a cyst with high protein/hematic content. Serology was also solicited for hydactidosis as a differential diagnosis and its result was negative.

After 3 months of the same, the patient complained of pain in the right hypochondrium with irradiation toward the right costal gradient and right shoulder, with worsening upon inhalation; she also referenced dyspnea associated with clinical pain, being hospitalized for controlling pain and diagnostic elucidation.

Given this presentation, at 63 months after the first surgery, the patient was referred to a surgical center to undergo exploratory laparotomy for enucleation of cystic lesions. However, in the intraoperative period it was possible to observe macroscopically lesioning in liver segments V, VI and VII, with biopsy being carried out by freezing of the lesion in segment V, which confirmed liver metastasis, with the surgeon opting to convert the surgery to right-sided hepatectomy, with removal of segments V, VI, VII and VIII. Immunohistochemistry of liver metastasis revealed expression positive for β catenin clone 14 and CD10 (CALLA, 56C6), findings consistent with solid pseudopapillary neoplasm (SPN) [10].

The patient was hospitalized for 27 days and then discharged from the hospital, after which she received monthly outpatient follow-up with clinical oncology and the general surgical team, without complaints and with negative tumor markers (Alpha-fetoprotein, CEA and CA 19.9), total abdominal CT without alterations.

Starting in May of 2017, the outpatient return consultations became quarterly with the surgical team and semiannual with oncology. Adjuvant chemotherapy was not performed while laboratory and imaging exams were maintained within normality patterns, totaling global survival of 100 months after the first treatment, the first surgery.

## 3 Discussion

This study showed a patient who at 13 years of age presented epigastric pain, weight loss and imaging findings suggestive of pancreatic tumor, with surgery being indicated. The literature highlights that SPN is most common in young women, with an average age of 30 years, and is rarer at 13 years [4,7,8].

She developed left hypochondrium pain and epigastric pain, palpable mass accompanied by nonspecific symptoms secondary to pancreatic compression (nausea and fever), similar to previous studies [7,9,10]. The tumor occupied the body and tail of the pancreas, but some studies cite the tail of the pancreas as the topography most commonly affected, followed by the head and body, or even manifesting synchronically in the head and tail of the pancreas [11].

The initial treatment of SPN is surgical, and it depends on the clinical-pathological characteristics of each patient, for which there is no standardization [4,8]. Some authors emphasize that intraoperative analysis being decisive related to differential diagnosis and histopathological and immunohistochemical exams [12]. Aggressive surgeries were associated with improved prognoses, revealing a divergence in the choice of surgical technique to be applied, in which macroscopic evaluations may favor incomplete resection, recurrence and distant metastasis [7].

In this study, the histopathological result evidenced disease limited to the pancreas, with capsule intact and hemorrhagic signals on the external surface of organs adhering to the pancreatic tumor mass. Furthermore, after 30 months, the patient evolved with multiple liver metastases, even in total tumor resection.

Local recurrence or distant metastasis is described in about 15% of SPN cases, with the liver being the most affected organ [8], associated with tumor rupture or invasion of an adjacent organ [7,13]. According to the American Joint Committee on Cancer (AJCC) and the WHO, angioinvasion, unequivocal perineural invasion and deep invasion of the present pancreatic parenchyma are histological criteria that indicate greater probability of recurrence or metastasis, classifying this as a tumor with uncertain malignant potential [6].

Here, the patient manifested liver images suggestive of metastasis at 30 months after surgery, diverging from the liver metastasis incidence (15%) more commonly described from the early form (between 3 and 17 months after initial surgery) [7,14].

Total tumor resection has ensured prolonged survival above 95% when SPN is limited to the pancreas or even curative in the resection of metastatic liver disease [4,8]. In addition, the survival times of 5 years even in metastatic disease and 10 years in total tumor resection exceed 90% [14]. Currently, the patient is in the outpatient segment without complaints correlated with the basic disease, and without laboratory or imaging alterations, totaling global survival of 100 months.

It is concluded that this case report is important for ratifying the prognostic and clinical polymorphism spectrum of SPN. Furthermore, it is understood to be a borderline tumor with indolent biological behavior, there are still multiple poorly understood factors that can dictate its malignant transformation and prognosis. Therefore, there is a need for a long and rigorous segment to identify possible long-term metastases. It is necessary to conduct additional epidemiological studies, and molecular and clinical trials that can augment the knowledge on TSP.

## 4 Conclusion

The present study followed the guidelines for case reports (SCARE) [15] and concluded some importants thinks. First, this case report is important for ratifying that the prognostic and clinical polymorphism spectrum of SPN. Furthermore, it is understood to be a borderline tumor with indolent biological behavior, there are still multiple poorly understood factors that can dictate its malignant transformation and prognosis. Therefore, there is a need for a long and rigorous segment to identify possible long-term metastases.

Secondly, the surgical procedure associated with regular and prolonged postoperative follow-up favored the diagnosis and resection of liver metastases in a timely manner, with positive impact on patient survival and quality of life. Currently, she is in the semi-annual and disease-free outpatient segment, with a total of 96 months overall survival. Lastly, it is necessary to conduct additional epidemiological studies, and molecular and clinical trials that can augment the knowledge on TSP.

## Declaration of Competing Interest

None.

## Sources of funding

Funded by the authors.

## Ethical approval

This case report was elaborated and developed according to the legal and ethical precepts of Resolution Nº466/12 from the National Health Council/Brazilian Ministry of Health and approved by the Committee for Ethics in Research in the Nucleus for Oncology Research under Protocol Nº 2.730.231.

## Consent

Written informed consent was obtained at 18 years old patient to publish this case report and accompanying images. A copy of the written consent is available for review by the Editor-in-Chief of this journal on request.

## Author contribution

Gisela: conception or design of the work and acquisition, Project administration; analysis data.

Aline: conception or design of the work and acquisition, Project administration; analysis data.

Ana: acquisition data.

Samia: drafting the work or revising it critically for important intellectual content.

Williams: interpretation of data for the work.

Helena: acquisition data.

Marta: acquisition data.

Amanda: acquisition data.

Geraldo: interpretation of data for the work.

Paulo: Data curation; Formal analysis, drafting the work or revising it critically for important, intellectual content; review & editing.

## Registration of research **s**tudies

**CAAE:** 90972818.6.0000.5634.

## Guarantor

Paulo Assumption and Aline Cruz.

## Provenance and peer review

Not commissioned, externally peer-reviewed.

## References

[1] Frantz V.K. (1959). Tumors of the pancreas. Atlas of Tumor Pathology, 1st Series, Fascicle 27-28.

[2] Crucitti A., Grossi U., Giustacchini P., Tomaiuolo P.M.C., Bellantone R. (2010). Solid pseudopapillary tumor of the pancreas in children: report of a case and review of the literature. Updates Surg..

[3] Lin M.Y.C., Stabile B.E. (2010). Solid pseudopapillary neoplasm of the pancreas: a rare and atypically aggressive disease among male patients. Am. Surg..

[4] Escobar M.A., Bond B.J., Schopp J. (2014). Solid pseudopapillary tumour (Frantz’s tumour) of the pancreas in childhood. BMJ Case Rep..

[5] McCluney S., Wijesuriya N., Sheshappanavar V., Chin-Aleong J., Feakins R., Hutchins R., Abraham A., Bhattacharya S., Valente R., Kocher H. (2018). Solid pseudopapillary tumour of the pancreas: clinicopathological analysis. ANZ J. Surg..

[6] (2013). International Classification of Diseases for Oncology (ICD-O).

[7] Carlotto J.R.M., Torrez F.R.A., Gonzalez A.M., Linhares M.M., Triviño T., Herani-Filho B., Goldenberg A., Lopes-Filho G.J., Lobo E.J. (2016). Solid pseudopapillary neoplasm of the pâncreas. ABCD Arq. Bras. Cir. Dig..

[8] Antoniou E.A., Damaskos C., Garmpis N. (2017). Solid pseudopapillary tumor of the pancreas: a single-center experience and review of the literature. In Vivo (Brooklyn).

[9] Dinarvand P., Lai J. (2017). Solid pseudopapillary neoplasm of the pancreas: a rare entity with unique features. Arch. Pathol. Lab. Med..

[10] Wu H., Huang Y.-F., Liu X.-H., Xu M.-H. (2017). Extrapancreatic solid pseudopapillary neoplasm followed by multiple metastases: case report. World J. Gastrointest. Oncol..

[11] Jakhlal N., Njoumi N., Hachi H., Bougtab A. (2016). Tumeur pseudopapillaire et solide du pancréas: à propos d’un cas et revue de la literature. Pan Afr. Med. J..

[12] Słowik-Moczydłowska Ż, Gogolewski M., Yaqoub S., Piotrowska A., Kamiński A. (2015). Solid pseudopapillary tumor of the pancreas (Frantz’s tumor): two case reports and a review of the literature. J. Med. Case Rep..

[13] Ren Z., Zhang P., Zhang X., Liu B. (2014). Solid pseudopapillary neoplasms ofthe pancreas: clinicopathologic features and surgical treatment of 19 cases. Int. J. Clin. Exp. Pathol..

[14] Cai Y.-Q., Xie S.-M., Ran X., Wang X., Mai G., Liu X.-B. (2014). Solid pseudopapillary tumor of the pancreas in male patients: report of 16 cases. World J. Gastroenterol..

[15] Agha R.A., Borrelli M.R., Farwana R., Koshy K., Fowler A., Orgill D.P., For the SCARE Group (2018). The SCARE 2018 statement: updating consensus Surgical CAse REport (SCARE) guidelines. Int. J. Surg..

